# Cross-Platform Evaluation of Commercial Real-Time SYBR Green RT-PCR Kits for Sensitive and Rapid Detection of European Bat *Lyssavirus* Type 1

**DOI:** 10.1155/2015/839518

**Published:** 2015-02-16

**Authors:** Evelyne Picard-Meyer, Carine Peytavin de Garam, Jean Luc Schereffer, Clotilde Marchal, Emmanuelle Robardet, Florence Cliquet

**Affiliations:** Anses, Laboratory for Rabies and Wildlife, WHO Collaborating Centre for Research and Management in Zoonoses Control, OIE Reference Laboratory for Rabies, European Union Reference Laboratory for Rabies, European Union Reference Laboratory for Rabies Serology, Technopôle Agricole et Vétérinaire, CS 40009, 54220 Malzéville, France

## Abstract

This study evaluates the performance of five two-step SYBR Green RT-qPCR kits and five one-step SYBR Green qRT-PCR kits using real-time PCR assays. Two real-time thermocyclers showing different throughput capacities were used. The analysed performance evaluation criteria included the generation of standard curve, reaction efficiency, analytical sensitivity, intra- and interassay repeatability as well as the costs and the practicability of kits, and thermocycling times. We found that the optimised one-step PCR assays had a higher detection sensitivity than the optimised two-step assays regardless of the machine used, while no difference was detected in reaction efficiency, *R* ^2^ values, and intra- and interreproducibility between the two methods. The limit of detection at the 95% confidence level varied between 15 to 981 copies/*µ*L and 41 to 171 for one-step kits and two-step kits, respectively. Of the ten kits tested, the most efficient kit was the Quantitect SYBR Green qRT-PCR with a limit of detection at 95% of confidence of 20 and 22 copies/*µ*L on the thermocyclers Rotor gene Q MDx and MX3005P, respectively. The study demonstrated the pivotal influence of the thermocycler on PCR performance for the detection of rabies RNA, as well as that of the master mixes.

## 1. Introduction

Rabies, which is caused by all members of the genus* Lyssavirus*, family Rhabdoviridae, is a viral encephalitis that is found on every continent except Antarctica. The* Lyssavirus* genus is divided into 14 species, the majority of which are predominantly associated with infection in bats [1]. Bats are natural reservoirs for all lyssaviruses and are considered to represent the ancestral host for the lyssaviruses [2]. In Europe, bat rabies is caused by four lyssaviruses, namely, European bat* Lyssavirus* type 1 (EBLV-1), European bat* Lyssavirus* type 2 (EBLV-2), Bokeloh bat* Lyssavirus* (BBLV), and Lleida bat* Lyssavirus* (LLEBV). Between 1977 and 2013, more than 1,000 cases of bat rabies were reported [3], with the majority (>97%) being attributed to EBLV-1.

Rabies diagnosis based upon clinical presentation or gross pathognomonic lesions is unreliable, because signs of the disease are not characteristic and may vary greatly from one animal to another. Therefore, diagnosis in animals relies on postmortem laboratory findings. The WHO “gold standard” test for rabies diagnosis in animals is the fluorescent antibody test (FAT) in which FITC anti-rabies virus antibody is applied to selected regions of the brain collected postmortem [4]. Evidence of a rabies infection can be demonstrated through the detection of antigen (FAT), infectious virus (rabies tissue culture infection test (RTCIT), mouse inoculation test (MIT)), or viral RNA (nucleic acid amplification tests), these conventional OIE diagnostic tests being entirely dependent on the nature and quality of the sample supplied. With the advent of molecular biology since the 1980s and due to the immense versatility and sensitivity of PCR, diagnostic methods based on PCR technology have been extensively applied to many infectious diseases [5]. To date, real-time PCR is the state-of-the-art technology for the quantification of nucleic acids. Various nucleic acid detection-based assays have thus been published for the detection of* Lyssavirus* fragments using conventional gel-based PCR, NASBA, or real-time PCR [6]. In Europe, most laboratories working on rabies employ conventional and/or real-time PCR [7] due to their high specificity and sensitivity. The conventional methods are reported to be very sensitive when using a heminested RT-PCR [8] and compared to heminested RT-PCR, real-time PCR is shown to be about 100–1,000-fold more sensitive [9]. However, due to this high PCR sensitivity, which engenders a risk of sample cross contamination and therefore a risk of potential false positive results, RT-PCR is not recommended by the World Health Organization for routine postmortem diagnosis of rabies in animals [4].

Real-time RT-PCR which provides rapid, sensitive, and specific detection of rabies virus decreases the risk of sample contamination. Various real-time assays have thus been described since 2004 [10] for the rapid detection of RABV or other* Lyssavirus* species [11, 12] using the one-step method [13, 14] or the two-step method [12, 15, 16]. Two systems of real-time assays are widely used for the detection of RABV and lyssaviruses: the SYBR Green real-time RT-PCR and the TaqMan real-time RT-PCR, which is to date the most common technique implemented in the laboratories working on rabies [17].

As real-time PCR assays have become routine in diagnostic laboratories and because of their prominence, there has been rapid growth of commercially available reagent systems and detection platforms which greatly increase the options available to laboratories. In this study, we evaluated several commercial master mixes in order to identify those which produce the best results using one-step and two-step real-time RT-PCR for the determination of EBLV-1. Five different systems for the generation of cDNA, five two-step PCR kits, and five one-step qRT-PCR kits were compared. The analysed performance evaluation criteria included the generation of a standard curve, reaction efficiency, analytical sensitivity, intra- and interassay repeatability as well as the costs and the practicability of kits, and thermocycling times. The goal of this study was also to determine if the one-step and two-step systems had the same reaction efficiencies for the detection of EBLV-1 and if one system was more sensitive than another using two real-time thermocyclers.

## 2. Materials and Methods

### 2.1. Rabies Virus Isolates and RNA Extraction

The European bat* Lyssavirus* type 1 virus (EBLV-1) was selected for this study for the generation of* in vitro* RNA transcript. The virus EBLV-1 (ANSES, strain numbered 05-09) was isolated in France in 2000 from a rabid bat (*Eptesicus serotinus*). The isolate was mouse passaged before RNA extraction.

Viral RNA was extracted from 200 *μ*L of 10% mouse brain tissue suspension using the Iprep Pure Link Virus kit (Invitrogen, France) according to the manufacturer's instructions. The RNA concentration was determined spectrophotometrically and the RNA was stored at −70°C until use.

### 2.2. Preparation of* In Vitro* RNA Transcripts

Briefly, a one-step RT-PCR amplification was performed as previously described [8] on 5 *µ*L of extracted RNA (i.e., 250–500 ng) with specific rabies primers (JW12 (forward) 5′-ATGTAACACCYCTACAATG and JW6 (reverse) 5′-CARTTVGCRCACATYTTRTG). The 606 bp PCR products were purified and inserted into a pGEM T easy vector (Promega, France) according to the manufacturer's instructions. Recombinant plasmids were purified using the PerfectPrep EndoFree Plasmid Maxi Kit (5Prime, France) and then concentrated using a step of sodium acetate/ethanol precipitation and linearised with the PstI restriction enzyme (Promega, France). The clones of PCR products were bidirectionally sequenced with sense and antisense primers T7 and SP6 by Beckman Coulter Genomics (Takeley, United Kingdom) to confirm the presence of the target insert.

*In vitro* transcription was performed using T7 RNA polymerase according to the manufacturer's instructions (MaxiScript SP6/T7 kit, Ambion, France). The resulting RNA transcripts were purified as recommended by the manufacturer using 2 *µ*L of DNAse I during 15 min at 37°C, followed by a final purification step using the RNeasy mini kit (Qiagen, France). The RNA concentration was determined by using the Qubit Fluorometer (Invitrogen, France). RNA transcripts were aliquoted to 10^7^ copies/*µ*L and then stored at −70°C. The RNA molecule copy number was calculated following the manufacturer's instructions (MaxiScript SP6/T7 kit, Ambion, France) using the following formula: *Y* molecules/*µ*L = (*X* g/*µ*L RNA/[transcript length in nucleotides × 330] × 6.022·10^23^). The estimated number of copies was of 2.68 · 10^11^ for EBLV-1 RNA transcript.

The quality of RNA transcript was checked on serial tenfold dilutions (10^6^ to 1 copie/*µ*L) through measurements of efficiency of amplification using a two-step SYBR Green RT-PCR [12]. PCR efficiency was of 99%.

### 2.3. Real-Time RT-PCR Assays

#### 2.3.1. Primers Used

Universal pan* Lyssavirus* primers were selected for this study. Two questionnaires, respectively, undertaken in 2011-2012 by the EURL laboratory have demonstrated that 13 out of 21 National Reference Laboratories for Rabies (NRLs) used primers JW12 and N165-146 previously described by Wakeley et al. [18]. These specific rabies primers (JW12 (forward), ATGTAACACCYCTACAATG and N165-146 (reverse), GCAGGGTAYTTRTACTCATA) were designed to detect the rabies virus N gene of all known lyssaviruses [12].

#### 2.3.2. Two-Step RT-qPCR Assays

*(1) Commercial Master Mix Kits*

*(a) Reverse Transcription Kits (Table 1(a))*. Five different reverse transcription (RT) systems provided by five different companies and five different qPCR kits provided by the same five suppliers were tested with the two-step method. The following RT kits were compared: Iscript (IScript cDNA synthesis kit, Bio-Rad, France), QuantiTect (Quantitect Reverse transcription kit, Qiagen, France), Maxima (Maxima H Minus First Strand cDNA synthesis kit, Fermentas, France), SS Vilo (Superscript Vilo Master Mix, Invitrogen, France), and qScript (qScript cDNA Synthesis kit, Quanta, France).

*(b) Two-Step SYBR Green PCR Kits (Table 1(b))*. Different two-step qPCR kits were tested: SsoFast (SsoFast Evagreen Super mix kit, Bio-Rad, France), QuantiTect (QuantiTect SYBR Green PCR kit, Qiagen, France), Maxima (Maxima SYBR Green qPCR master mix, Fermentas, France), Express (Express SYBR GreenER qPCR Supermix universal, Invitrogen, France), and Perfecta (Perfecta SYBR Green Supermix, Quanta, France).

*(2) Two-Step RT-qPCR Analysis*. Seven 1 : 10 serial dilutions (i.e., 10^6^ to 1 copie/*µ*L) of the* in vitro* EBLV-1 RNA transcript were prepared in RNAse free water then subjected to reverse transcription using the five different RT master mixes. All RT kits were optimised with regard to the time and the temperature of incubation (Table 1(a)). Complementary cDNAs were generated from 5 *µ*L of each dilution of EBLV-1 RNA, random primer, oligo dT, or a mix of oligo dT/random primer included in each tested kit according to the recommendations of the manufacturers. The cycling conditions and the reaction protocol for each tested RT kit are detailed in Table 1(a). Thermocycling was performed on the Mastercycler Eppendorf thermal cycler (Eppendorf, France).

Real-time PCRs were run on the thermocyclers Rotor Gene Q MDx (Qiagen, France) and Mx3005P (Agilent Technologies, France). The concentrations of the reverse (N165-146) and forward (JW12) primers were optimised for each tested kit. At least three different concentrations were examined, that is, the one recommended by the manufacturer, one below, and one above the recommended primer concentration using the qPCR conditions described in Table 1(b). The optimised concentration of primers is recorded for each tested qPCR kits in Table 1(b). Reactions were performed in triplicate in a total volume of 20 *µ*L or 25 *µ*L, following the manufacturer's instructions (Table 1(b)).

The following thermal profile was used: PCR initial activation step at 95°C for 30 sec (SsoFast), 15 min (QuantiTect), 10 min (Maxima), 2 min (Express), or 3 min (Perfecta) according to the manufacturer's recommendations. The cycler conditions, performed as previously described by Hayman et al. [19], were the same for all assays: 45 cycles of three-step cycling consisting of denaturation at 94°C for 30 sec, annealing at 55°C for 30 sec, and extension at 72°C for 30 sec.

#### 2.3.3. One-Step qRT-PCR Assays

*(1) Commercial qRT-PCR Kits (Table 1(c))*. Five commercial one-step kits provided by four suppliers were tested: QuantiTect-1 step (QuantiTect SYBR Green RT-PCR kit, Qiagen, France), Rotor Gene-1 step (Rotor-Gene SYBR Green RT-PCR kit, Qiagen, France), Verso-1 step (Verso SYBR Green one-step qRT-PCR kit, Fermentas, France), SSIII Platinium-1 step (SuperScript III Platinium SYBR Green One-step qRT-PCR kit, Invitrogen, France), and qScript-1 step (qScript One step SYBR Green QRT-PCR kit, Quanta, France).

*(2) One-Step qRT-PCR Analysis*. To compare the five master mixes, the assays were performed in triplicate using seven tenfold dilutions (i.e., 10^6^ to 1 copies/*µ*L) of EBLV-1 RNA. PCR was performed using a total volume of 25 *µ*L containing 2 *µ*L of serial dilutions of EBLV-1 RNA and the master mix components (*V* = 23 *µ*L) from each representative kit, which were prepared according to the respective manufacturer's instructions (Table 1(c)). The concentrations of the reverse (N165-146) and forward (JW12) primers were optimised for each tested kit (Table 1(c)). At least three different concentrations were examined, the one recommended by the manufacturer, one below, and one above the recommended primer concentration (Table 1(b)).

Real-time RT-PCR assays were performed on the thermocycler Rotor Gene Q MDx and Mx3005P using the same thermocycling conditions for the five kits (i.e., 45 cycles of 30 sec-94°C, 30 sec-55°C and 30 sec-72°C). For each tested kit, the DNA polymerase activation step was performed as recommended by the supplier (Table 1(c)).

#### 2.3.4. Data Collection and Analysis

PCR efficiency and coefficient of determination (*R* ^2^) values were calculated by the software Rotor Gene Q Series and Mx3005Pro, respectively.

Negative and positive controls were included in each assay, in which a threshold setting (Ct) of 0.03 was used as the reference for the Rotor Gene Q MDx thermocycler. A threshold setting of 300 was used as the reference for the Mx3005P thermocycler for all tested kits except for one two-step kit (Express) for which a threshold setting of 0.05 was used.

### 2.4. Determination of the Performance of the Method

The performance of the method was determined for all tested kits following OIE recommendations [20] and those of the French Agency for Standard Operating Procedure for PCR experimental design [21]. Performance testing was conducted for the two real-time platforms.

#### 2.4.1. Determination of PCR Efficiency

A 10^6^ to 10^3^ copies/*µ*L dilution range corresponds to the linear dynamic range for the ten tested kits. To determine the PCR efficiency for each tested kit, a standard curve was plotted based on Ct values obtained from samples containing 10^6^ to 10^3^ copies/*µ*L against the log of the estimated RNA copy number in the sample. The coefficient of determination (*R* ^2^) was calculated and considered as suitable when not lower than 0.99. The slope of the standard curves was used for the determination of PCR efficiency (*E* = 10[−1/slope] − 1). The efficiency was considered satisfactory when it is not lower than 90% and not above 110% [22].

#### 2.4.2. Determination of the Limit of Detection at 95%

The limit of detection at 95% (LOD 95%) which corresponds to the lowest RNA concentration that could be detected with 95% confidence was calculated using the inverse standard normal distribution calculation [21]. The LOD 95% was calculated using the Probit analysis based on the results of three runs of eight repetitions of six dilutions each of EBLV-1 RNA in RNase-free water (*n* = 144 samples tested). The six dilutions (cDNA and RNA) were selected surrounding the limit of detection of real-time PCR assays.

On the Rotor Gene Q MDx gene cycler, 10^3^ to 25 copies/sample were tested with the kit Rotor Gene-1 step, 10^3^ to 50 copies/sample for qScript-1 step/SSIII Platinium-1 step, 500 to 10 copies/sample for Verso-1 step, 50 to 1 copies/sample for Quantitect-1 step, 250 to 5 copies/sample for Perfecta, 250 to 5 copies/sample for QuantiTect, 10^2^ to 2.5 copies/sample for Maxima, 100 to 0.5 copies/sample for Sso Fast, and 100 to 0.5 copies/sample for Express.

On Mx3005P cycler, 2500 to 100 copies/sample were tested with the kit qScript-1 step, 250 to 5 copies/sample for SSIII Platinium-1 step/Verso-1 step, 50 to 1 copies/sample for Rotor Gene-1 step/Quantitect-1 step, 500 to 10 copies/sample for QuantiTect, 250 to 5 copies/*µ*L for Maxima/Sso Fast/Perfecta, 250 to 5 copies/sample for Perfecta, and 100 to 2.5 copies/sample for Express.

Three independent assays were undertaken under identical laboratory conditions.

#### 2.4.3. Evaluation of Reproducibility

To evaluate the reproducibility of the ten kits, inter- and intraspecific assay reproducibility were assessed using the coefficient of variation (CV) of the Ct values for the lowest RNA concentration showing 100% positive results. The intra-assay repeatability was determined using three independent runs of eight replicates of EBLV-1 (*n* = 24 samples tested). The interassay repeatability was assessed for the same serial replicates in three independent assays using identical laboratory conditions. CVs expressed as percentages were obtained by dividing the standard deviation of each tested sample by the mean of the cycle threshold values and multiplying that result by 100.

## 3. Results

### 3.1. Differences in RNA Detection Levels Using Two-Step Assays

#### 3.1.1. Reverse Transcription Kits

Of the five reverse transcription kits tested in combination with the SSoFast kit for cDNA amplification, the SuperScript Vilo yielded greater sensitivity and resulted in a detection limit of 10 copies/*µ*L (3 positive samples out of 3 tested). The efficiency of amplification was of 98%. The four other RT kits (i.e., kits Quantitect, Maxima, SS Vilo, and qScript) resulted in a detection of 100 copies/*µ*L. The amplification efficiency varied between 98 and 104%. The *y*-intercept for all reverse transcription kits tested in combination with the qPCR SsoFast kit varied between 35.13 and 37.46.

#### 3.1.2. Two-Step PCR Kits

All optimised two-step qPCR assays tested in combination with the Iscript RT showed similar sensitivity and enabled detection of at least 100 copies/*µ*L of EBLV-1 on the Rotor Gene Q MDx gene cycler. The efficiency of amplification which was satisfactory for all tested kits varied between 90 (i.e., kit Perfecta) and 99% (i.e., kits SSo Fast, Quantitect SG, Maxima SG, and Express) with an *y*-intercept varying between 36.03 and 38.57.

#### 3.1.3. Comparison of Two-Step RT-qPCR Kits Provided by the Same Manufacturer

Table 2 details the results of the comparison of the five two-step kits.

*(i) Rotor Gene Q MDx*. Of the five PCR kits tested in combination with the reverse transcriptases provided by the same manufacturer, one RT-qPCR assay (SuperScript Vilo in combination with qPCR Express kit) yielded greater sensitivity and showed a detection of at least 10 copies/*µ*L (Table 2). All amplicons investigated with the two-step method using qPCR kits and RT kits from the same provider showed satisfactory efficiency of amplification (100 ± 10%). The *y*-intercept for all tested kits varied between 34.73 and 39.55.

*(ii) Mx3005P*. Of the five tested RT-qPCR kits, one RT-qPCR assay (Maxima RT/qPCR Maxima) yielded greater sensitivity and showed a detection of at least 10 copies/*µ*L. All RT-qPCR assays showed satisfactory efficiency varying between 93 and 107% with detection of at least 100 copies/*µ*L. The *y*-intercept varied between 38.01 and 41.83 for all tested kits.

### 3.2. Differences in RNA Detection between the Five Kits Tested Using the One-Step Method

Table 3 details the results of the comparison of the five one-step kits.

*(i) Rotor Gene Q MDx*. Three optimised qRT-PCR assays revealed poor sensitivity and enabled detection of at least 10^3^ copies/*µ*L. Of the five kits tested, two kits (QuantiTect-1 step and Verso-1 step) yielded greater sensitivity with the detection of at least 10 copies/*µ*L (3 positive samples out of 3 tested). All kits showed a satisfactory efficiency varying between 94% and 104%. The *y*-intercept varied between 32.78 and 37.67 for the five tested one-step kits.

A high presence of Primer-Dimer was shown for low RNA concentrations for three kits: SSIII Platinium-1 step, Verso-1 step, and qScript-1 step.

*(ii) Mx3005P*. Of the five one-step kits tested using the Mx3005P cycler, three optimised one-step kits (i.e., Quantitect-1 step, Rotor Gene-1 step, and SSIII Platinium-1 step) revealed good sensitivity with the detection of at least 10 copies/*µ*L. The two other optimised one-step assays (i.e., Verso-1 step and qScript-1 step) revealed poor sensitivity with a detection of 10^2^ and 10^3^ copies/*µ*L, respectively. The amplification efficiency was satisfactory for all kits (94–100%) except for the qScript one-step kit (efficiency of 87%). The *y*-intercept varied between 36.80 and 40.74 for all tested kits except for the qScript kit (Intercept of 40.74).

### 3.3. Comparison of Assay Performance

#### 3.3.1. Limit of Detection at 95% for One-Step and Two-Step Methods

The results of LOD 95% for the ten kits tested using two-step and one-step methods are recorded in Table 4.

*(i) Rotor Gene Q MDx*. The results showed an LOD 95% for two-step and one-step kits ranging from 41 to 171 copies/*µ*L and from 20 to 497 copies/*µ*L, respectively (Table 4). Of the five commercial master mixes tested using the two-step qPCR, the RT SuperScript Vilo in combination with the Express kit yielded greater sensitivity with an LOD 95% of 41 copies/*µ*L. Maxima in combination with the RT Maxima and Perfecta in combination with the RT qScript ranked second and third with detection of 78 and 88 copies/*µ*L, respectively, followed by the SsoFast and QuantiTect kits which demonstrated the poorest levels of detection with an LOD 95% of 110 and 171 copies/*µ*L, respectively.

In contrast, the one-step qRT-PCR kit Quantitect-1 step yielded the best levels of detection with an LOD 95% of 20 copies/*µ*L. The four other tested one-step kits yielded poor detection with an LOD 95% varying between 127 and 497 copies/*µ*L.

*(ii) Mx3005P*. The LOD 95% for one-step and two-step kits ranged from 15 to 981 copies/*µ*L and 45 to 160 copies/*µ*L, respectively. Of the five commercial master mixes tested using the two-step qPCR, the RT SuperScript Vilo in combination with the Express kit yielded greater sensitivity with an LOD 95% of 45 copies/*µ*L. Perfecta in combination with qScript RT and Maxima in combination with RT Maxima ranked second and third, with a detection of 67 and 76 copies/*µ*L respectively, followed by the the SsoFast and QuantiTect kits which demonstrated the poorest levels of detection with an LOD 95% of 88 and 160 copies/*µ*L, respectively.

Of the five tested one-step kits, two kits (i.e., Rotor gene-1 step and Quantitect-1 step) yielded the best levels of detection with an LOD 95% of 15 and 22 copies/*µ*L, respectively. SSIII Platinium-1 step ranked third with an LOD 95% of 50 copies/*µ*L followed by Verso-1 step (LOD 95% of 101 copies/*µ*L) and qScript-1 step (981 copies/*µ*L).

#### 3.3.2. Reproducibility of One-Step and Two-Step Methods

Table 5 shows the analysis results of reproducibility of one-step and two-step assays.

*(i) Rotor Gene Q MDx*. For all optimised one-step or two-step PCR assays, the interassay CVs were lower than 10%, demonstrating good reproducibility for all tested kits [23]. The interassay CVs for the two-step kits and the one-step kits ranged from 1.74 to 2.95 and 1.35 to 4.27, respectively, while the intra-assay CVs ranged from 1.35 to 3.5 and 0.79 to 6.72 for two-step and one-step kits, respectively (Tables 5(a) and 5(b)). In analysing the intra-assay CVs, a somewhat similar variability was shown for two-step and one-step reactions.

The interassay CVs for the most efficient two-step kit (i.e., SuperScript Vilo in combination with qPCR Express) and the most efficient one-step kit (i.e., Quantitect-1 step) ranged from 1.95 to 3.36, respectively, while the intra-assay CVs ranged from 1.85 to 2.11 and 0.79 to 2.16 for two-step and one-step kits, respectively (Tables 5(a) and 5(b)).

*(ii) Mx3005P*. For all optimised one-step or two-step PCR assays, the interassay CVs were lower than 10%, demonstrating good reproducibility for all tested kits. The interassay CVs for the two-step and one-step kits ranged from 2.14 to 3.3 and 0.84 to 4.5, respectively, while the intra-assay CVs ranged from 1.23 to 4.22 and 0.38 to 4.7 for two-step and one-step kits, respectively.

### 3.4. Practicability of the Different Assays, Costs, and Time

Table 6 shows a practicability assessment of the different real-time assays including a comparison of cost, pipetting steps, and thermocycling times. Total reaction times for the ten real-time assays ranged from 2h42 to 3h08 for two-step kits and 2h18 to 2h57 for one-step kits, with the one-step SSIII Platinium kit requiring the shortest time, and two-step Maxima kit the longest, regardless the machine used. The kits were ranked from 1 to 10 based on the cost per PCR reaction in increasing order, from €1.08 (least expensive = 1) to €12.25 (most expensive = 10). Overall, of the 10 master mix kits tested, the one-step kits were cheaper than the two-step kits (Table 6). For the one-step kits, qScript was the least costly followed by the Rotor Gene-1 step, Verso-1 step, SSIII Platinium-1 step, and the Quantitect-1 step, which was the most expensive of all the tested kits. The Express master mix kit with SuperScript Vilo was the most expensive per assay of the two-step kits evaluated in this study. Of the two-step kits, the least expensive was Perfecta, followed by Maxima, QuantiTect, and Sso Fast.

## 4. Discussion

The first practical real-time PCR, the 5′ nuclease assay, was established in 1993 combining the exponential PCR amplification of a specific transcript with the monitoring of newly synthesised DNA in each PCR cycle performed [24]. Over the past several years, real-time PCR has become the leading tool for the detection and quantification of nucleic acids. Since the first publications of real-time RT-PCR describing the rapid detection of rabies virus in 2004 [10, 25], various protocols for molecular diagnosis of* Lyssavirus* using SYBR Green and/or TaqMan probes have been published [11, 12, 16, 26, 27]. Although molecular techniques are not currently recommended by the international authorities for routine diagnosis of rabies, real-time PCR is increasingly used. The availability of commercial master mix kits has increased over the past decade, and diagnostic laboratories willing to implement real-time PCR are now faced with identifying the most suitable kit for their real-time PCR applications. It has been demonstrated that the choice of master mix reagents, the concentration of reaction mix components, such as primers, probes, analytical platforms linked to the real-time detection platform, the different chemistries, and the methodology used (one-step versus two-step systems) can impact the sensitivity and consistency of results for real-time PCR assays [28].

The objective of this study was to evaluate the sensitivity, efficiency, and reproducibility of one-step and two-step systems for the detection of EBLV-1. This virus species was selected for this study for the generation of* in vitro* RNA transcript. This bat virus is widely isolated on European bats, the vast majority of rabies cases in bats in Europe being attributed to EBLV-1. The increased interest in bats as a potential host for zoonotic viruses has resulted in the detection of many new* Lyssavirus* species in the past ten years [29]. In our study, we selected five different cDNA generation systems and ten commercial kits, that is, five one-step kits and five two-step kits. These kits are commonly used by National Reference Laboratories that conduct rabies diagnosis (data not shown). Our results demonstrated that there was little difference in reaction efficiency, *R* ^2^ values, or intra- and interreproducibility between one-step and two-step methods. In our experience, regardless of the real-time cycler used, one-step qRT-PCR showed better limit of detection at 95% than the optimised two-step assays using pan* Lyssavirus* primers.

The RT reaction is a key step in quantitative two-step RT-qPCR. RT priming strategy, dynamic range, and the RT enzyme, which are prime considerations for a successful real-time RT-PCR assay, are all important for ensuring that mRNA expression levels are accurately represented in the resulting cDNA [30]. For many quantitative applications, the MMLV RT (Moloney Murine Leukemia Virus reverse transcriptase) is described as the enzyme of choice as its cDNA synthesis rate is up to 40-fold greater than that of AMV (Avian Myeloblastosis Virus) reverse transcriptase [23]. Few results comparing the sensitivity of reverse transcriptases are currently available [31]. In this study, five different cDNA generation systems were compared (e.g., 4 different MMLV RTs and a mix of Omniscript and Sensiscript RTs), each kit containing unspecific target gene primers (e.g., random hexamer (*n* = 3 kits), a mix of random hexamers, and oligo dT (*n* = 2 kits)). By varying the RT chemistry and the RT priming strategy, we showed that the SuperScript Vilo reverse transcriptase which is an engineered version of MMLV RT has provided the highest sensitivity with a gain of 1 log over the other systems of cDNA generation. These results are concordant with those of the literature [23, 32, 33], demonstrating the key role of the reverse transcriptase kit in a sensitive real-time two-step assay and providing the highest MMLV reverse transcriptase performance.

In addition to assay performance criteria, another goal of this study was to compare the performance of each kit with the cost, the practicability of each tested master mix, and the presence or absence of Primer-Dimer for low DNA concentrations. Interestingly, the one-step QuantiTect SYBR Green RT-PCR kit, which was shown the more efficient one-step kits, is also one of the most expensive and required the longest thermocycling time while the combination of SuperScript VILO reverse transcriptase and the qPCR Express kit which was found to be the most efficient of the two-step kits using the two thermocyclers is also the most expensive of the two-step kits. No difference in run duration was shown between the best two-step kits, SuperScript VILO/qPCR Express and Maxima H Minus RT/qPCR Maxima, and the one-step kit, QuantiTect SYBR Green RT-PCR, confirming that all the kits are suitable for RNA detection, regardless of the thermocycling times. However, when using the thermocycler Rotor Gene MQDx, the real-time one-step SYBR Green assays with universal pan* Lyssavirus* revealed a high presence of Primer-Dimers for three out of five one-step kits tested especially when RNA concentration was low (below 100 copies/*µ*L) compared to two-step kits (data not shown). The results of such techniques should be interpreted with attention, since interpretation can be difficult, especially for low concentrations of samples.

Few studies have been performed comparing one-step and two-step methods, the chemistries (Taqman probes versus SYBR green) or the platforms for detection of infectious agents. There are advantages and disadvantages to both methods, as well as to the use of SYBR green or fluorogenic probes. The main advantage of the one-step method is that it is less time consuming and requires no user intervention, therefore minimising the chance of pipetting errors and cross contamination. The main advantage to two-step RT-qPCR is that typically random hexamer or oligo dT primers are used in an RT reaction in a separate tube allowing the archiving of samples for further testing of different species of rabies or for future testing of other genes. Moreover, in theory the one-step method should offer higher or at least the same sensitivity as a two-step method because of the use of gene-specific primers. In our experience, of the five one-step method kits tested, we found three that yielded satisfactory analytical sensitivity with an LOD 95% of 15–50 copies/*µ*L. Our results are concordant with previous results undertaken by Fischer et al. [32]. Based on the analysis of Ct values, a study of real-time RT-PCRs on infected brain samples [32] showed that three one-step SYBR Green kits were more sensitive than the two two-step kits.

The sensitivity of real-time PCR assays is mainly determined by the master mix reagents, but also by other factors, such as the platform used. The performance of the thermocycler has a critical influence on PCR efficiency. Previous studies have reported significant variability in PCR efficiency and results among different types of thermocyclers [34–36]. Considering the various types of real-time equipment used in National Reference Laboratories involved in rabies diagnosis, the ten master mixes were evaluated for intermediate reproducibility on two real-time platforms with varying throughput capacities. These two platforms differ on thermal engine (heating block technology based on the Peltier effect versus heat exchange technology which permit more rapid thermal ramp rates than blocks). Both real-time platforms are currently used by half of the National Reference Rabies laboratories (i.e., 9/19). We showed that the more efficient one-step kit was the one-step QuantiTect SYBR Green RT-PCR kit with an LOD 95% of 20 and 22 copies/*µ*L on the two real-time cyclers “Rotor Gene Q MDx, Mx3005P,” respectively. The reverse transcription kit SuperScript VILO in combination with the EXPRESS SYBR GreenER kit is revealed to be the most sensitive of the two-step kits offering a limit of detection of 41 and 45 copies/*µ*L, respectively. Previous studies have reported the influence of the thermocyclers. Buzard et al. demonstrated that certain combinations of commonly used master mixes and instruments are not as reliable as others for the detection of low DNA concentrations [28]. In our experiment, the LOD 95% of Rotor Gene-1 step from QIAGEN operated with the Rotor Gene Q MDX thermocycler was 497 copies/sample whereas it resulted in 15 copies/sample with the Mx3005P thermocycler. Our results demonstrated the pivotal influence of the thermocycler on PCR performance and that a master mix that showed high performance for one machine may not necessarily work on another. Our findings further show that PCR protocols need to be optimised for different instruments and that validation of PCR protocols should include the intermediate reproducibility studies as recommended by the World Organization for Animal Health [37].

PCR protocols need to be validated, since a large variety of parameters can influence the outcome of PCR amplification. PCR results generated by one lab are hardly reproducible by another, even under identical assay conditions. Due to the high sensitivity of real-time PCR assays, if they are not performed by highly trained molecular biology staff, there is a high risk of sample or test contamination, resulting in false positives. For these reasons, although these molecular tests have the highest level of sensitivity, their use is currently not recommended for routine postmortem diagnosis of rabies [4] due to the high levels of false positive or false negative results [7]. The considerable development of molecular techniques in the 2000s has resulted in a wide variation in methods and these techniques have become increasingly important for decision-making in rabies case management. However, it is essential that laboratories employing PCR-based assays apply very stringent laboratory quality control standards and that they validate these highly sensitive techniques following international guideline recommendations [37].

## 5. Conclusion

With the optimisation of PCR on two real-time instruments as well as the optimization of the RT step when testing the two-step method, we showed that one-step PCR is more sensitive than the two-step method, regardless of the machine used. We showed that the more efficient one-step kit was the one-step QuantiTect SYBR Green RT-PCR kit with an LOD 95% of 20 and 22 copies/*µ*L on the two real-time cyclers “Rotor Gene Q MDx, Mx3005P,” respectively. PCR assays demonstrated the crucial influence of the thermocycler on PCR performance for the detection of EBLV-1, despite the optimisation of PCR assays, and that a master mix which performs well on one machine may not necessarily work as well on another. In conclusion, PCR protocols should be systematically optimised for different instruments and validated following the recommendations of the World Organization for Animal Health.

## Figures and Tables

**Table:** (a) Reactions mix and thermocycling conditions for the five RT kits

Manufacturer	Reverse transcription kit	RTase and primers	Thermocycling conditions	Reaction mix^**^
Bio-Rad	Iscript select cDNA synthesis kit	M-MLV RT^*^ Random primer	5 min-25°C 30 min-42°C 5 min-85°C	4 *µ*L Iscript Mix (5x) + 2 *µ*L Random primer + 1 *µ*L Iscript RTase + 8 *µ*L RNase free water

Qiagen	QuantiTect Reverse Transcription Kit	Quantiscript RTase Mix OligodT + random primer	2 min-42°C 30 min-42°C 3 min-95°C	2 *µ*L gDNA Wipeout Buffer (7x) + 4 µL Quantiscript RT Buffer + 1 µL Quantiscript RTase + 1 µL RT primer + 7 µL RNase free water

Fermentas	Maxima H Minus First Strand cDNA Synthesis Kit	M-MLV RT^*^ Random primer	5 min-65°C 10 min-25°C 30 min-50°C 5 min-85°C	4 µL RT Buffer (5x) + 1 µL Random primer + 1 µL dNTP (10 mM) + 1 µL Maxima H Minus Ez Mix + 8 µL RNase free water

Invitrogen	SuperScript Vilo Master Mix	M-MLV RT^*^ Random primer	10 min-25°C 30 min-42°C 5 min-85°C	4 µL SuperScript Vilo MasterMix (5x) + 11 µL RNase free water

Quanta	qScript cDNA Synthesis Kit	M-MLV RT^*^ Mix OligodT + random primer	5 min-22°C 30 min-42°C 5 min-85°C	4 µL qScript Reaction Mix (5x) + 1 *µ*L qScript RTase + 10 *µ*L RNase free water

^*^Moloney Murine Leukemia Virus.

^**^Total volume of reaction is 20 *µ*L containing 5 *µ*L of RNA sample and 15 *µ*L of reaction mix. All RT assays were performed on the Mastercycler Eppendorf thermal cycler.

RTase: reverse transcriptase.

**Table:** (b) Reactions mix and thermocycling conditions for the five qPCR SybrGreen kits (two-step)

Manufacturer	qPCR SybrGreen kit	Thermocycling conditions	Reaction mix^*^
Bio-Rad	SsoFast EvaGreen Supermix	30 sec-95°C 45 cycles of 30 sec-95°C; 30 sec-55°C; 30 sec-72°C Melt Curve (60°C to 95°C)	Total volume of 20 *µ*L containing 2 *µ*L of cDNA and 18 *µ*L of a reaction mix (1x Sso Fast master mix, 0.35 *µ*M [0.5 *µ*M] each of *pan-Lyssavirus* primers)

Qiagen	QuantiTect SYBR Green PCR Kit	15 min-95°C 45 cycles of 30 sec-95°C; 30 sec-55°C; 30 sec-72°C Melt Curve (60°C to 95°C)	Total volume of 25 *µ*L containing 2 *µ*L of cDNA and 23 *µ*L of reaction mix (1x Quantitect Master mix, 0.5 *µ*M [0.6 *µ*M] each of *pan-Lyssavirus* primers)

Fermentas	Maxima SYBR Green qPCR Master Mix	10 min-95°C 45 cycles of 30 sec-95°C; 30 sec-55°C; 30 sec-72°C Melt Curve (60°C to 95°C)	Total volume of 25 *µ*L containing 2 *µ*L of cDNA and 23 *µ*L of reaction mix (1x Maxima SYBR Green master mix, 0.4 *µ*M [1 *µ*M] each of *pan-Lyssavirus* primers)

Invitrogen	Express SYBR GreenER qPCR Supermix Universal	2 min-95°C 45 cycles of 30 sec-95°C; 30 sec-55°C; 30 sec-72°C Melt Curve (60°C to 95°C)	Total volume of 20 *µ*L containing 2 *µ*L of cDNA and 18 *µ*L of reaction mix (1x Express SYBR GreenER Supermix, 0.4 *µ*M [2 *µ*M] each of *pan-Lyssavirus* primers) [50 nM of ROX dye]

Quanta	Perfecta SYBR Green SuperMix	3 min-95°C 45 cycles of 30 sec-95°C; 30 sec-55°C; 30 sec-72°C Melt Curve (60°C to 95°C)	Total volume of 25 *µ*L containing 2 *µ*L of cDNA and 23 *µ*L of reaction mix (1x Perfecta SYBR Green Supermix, 0.3 *µ*M [1 *µ*M] each of *pan-Lyssavirus* primers)

^*^The PCR reaction protocol corresponds to PCR assays performed on the Rotor Gene Q MDx. The concentrations of primers used on the real time thermocycler Mx3005P are shown in brackets [ ].

**Table:** (c) Reactions mix and thermocycling conditions for the five qRT-PCR SYBR Green kits (one-step)

Manufacturer	qRT-PCR SybrGreen kit	Thermocycling conditions	Reaction mix^*^
Qiagen	QuantiTect SYBR Green RT-PCR Kit	30 min-50°C, 15 min-95°C 45 cycles of 30 sec-95°C; 30 sec-55°C; 30 sec-72°C Melt Curve (60°C to 95°C)	1x QuantiTect SYBR Green master mix, 1% Quantitect RT mix, 0.6 *µ*M [2 *µ*M] each of *pan-Lyssavirus* primers

Qiagen	Rotor Gene-1 step: Rotor-Gene SYBR Green RT-PCR Kit	30 min-50°C, 5 min-95°C 45 cycles of 30 sec-95°C; 30 sec-55°C; 30 sec-72°C Melt Curve (60°C to 95°C)	1x Rotor-Gene master mix, 1% Rotor-Gene RT mix, 1 *µ*M [2 *µ*M] each of *pan-Lyssavirus* primers

Fermentas	Verso 1-step QRT-PCR Kit	30 min-50°C, 15 min-95°C 45 cycles of 30 sec-95°C; 30 sec-55°C; 30 sec-72°C Melt Curve (60°C to 95°C)	1x Verso SYBR Green master mix, 5% RT enhancer, 1% Verso enzyme mix, 0.2 *µ*M [0.3 *µ*M] each of *pan-Lyssavirus* primers

Invitrogen	SuperScript III Platinum SYBR Green One-Step qRT-PCR Kit	10 min-50°C, 5 min-95°C 45 cycles of 30 sec-95°C; 30 sec-55°C; 30 sec-72°C Melt Curve (60°C to 95°C)	1x SuperScript III Platinium master mix, 2% enzyme mix, 0.3 *µ*M [0.5 *µ*M] each of *pan-Lyssavirus* primers

Quanta	qScript One-Step SYBR Green RT-qPCR Kit	30 min-50°C, 5 min-95°C 45 cycles of 30 sec-95°C; 30 sec-55°C; 30 sec-72°C Melt Curve (60°C to 95°C)	1x qScript master mix, 2% qScript enzyme mix, 0.4 *µ*M [0.2 *µ*M] each of *pan-Lyssavirus* primers

^*^Total volume of reaction is 25 *µ*L containing 2 *µ*L of RNA sample and 23 *µ*L of reaction mix. The PCR reaction protocol corresponds to PCR assays performed on the Rotor Gene Q MDx. The concentrations of primers used on the real time thermocycler Mx3005P are shown in brackets [ ].

**Table 2:** Comparison of RT-qPCR SybrGreen kits (two-step) provided by the same manufacturer. Results of five different qPCR kits on a range of seven triplicate dilutions of EBLV-1 RNA.

Testing of different RT-qPCR kits (two-step) provided by the same manufacturer^****^											
Number of copies/sample	Number of copies/rxn	IScript RT + qPCR SsoFast		QuantiTect RT + qPCR Quantitect		Maxima RT + qPCR Maxima		SS Vilo RT + qPCR Express		qScript RT + qPCR Perfecta	
		Average Ct^***^	CV (%)^*^	Average Ct^***^	CV (%)^*^	Average Ct^***^	CV (%)^*^	Average Ct^***^	CV (%)^*^	Average Ct^***^	CV (%)^*^
10^6^	5.0 10^5^	*16.58 ± 0.13 *	*0.77 *	*19.47 ± 0.07 *	*0.34 *	*16.50 ± 0.15 *	*0.92 *	*15.01 ± 0.05 *	*0.35 *	*16.09 ± 0.03 *	*0.19 *
		**18.96 ± 0.04**	**0.21**	**22.79 ± 0.26**	**1.15**	**17.47 ± 0.09**	**0.52**	**17.49 ± 0.10**	**0.54**	**17.99 ± 0.29**	**1.59**
10^5^	5.0 10^4^	*19.93 ± 0.09 *	*0.45 *	*22.89 ± 0.10 *	*0.42 *	*19.50 ± 0.06 *	*0.33 *	*18.21 ± 0.05 *	*0.26 *	*19.79 ± 0.03 *	*0.13 *
		**22.17 ± 0.06**	**0.25**	**25.94 ± 0.09**	**0.35**	**21.16 ± 0.29**	**1.38**	**20.95 ± 0.03**	**0.15**	**21.27 ± 0.15**	**0.72**
10^4^	5.0 10^3^	*23.28 ± 0.05 *	*0.20 *	*25.89 ± 0.06 *	*0.25 *	*22.95 ± 0.28 *	*1.23 *	*21.68 ± 0.17 *	*0.76 *	*23.18 ± 0.16 *	*0.67 *
		**25.75 ± 0.05**	**0.18**	**29.60 ± 0.42**	**1.43**	**24.29 ± 0.29**	**0.39**	**24.40 ± 0.12**	**0.49**	**24.55 ± 0.08**	**0.33**
10^3^	5.0 10^2^	*26.61 ± 0.21 *	*0.80 *	*29.64 ± 0.32 *	*1.07 *	*26.43 ± 0.26 *	*0.98 *	*24.81 ± 0.05 *	*0.19 *	*26.94 ± 0.08 *	*0.30 *
		**29.16 ± 0.23**	**0.80**	**32.10 ± 0.19**	**0.60**	**27.78 ± 0.30**	**1.09**	**28.03 ± 0.74**	**2.63**	**27.84 ± 0.12**	**0.42**
10^2^	5.0 10^1^	*30.45 ± 0.13 *	*0.42 *	*32.20 ± 0.41 *	*1.26 *	*30.59 ± 0.45 *	*1.46 *	*28.33 ± 0.64 *	*2.25 *	*31.40 ± 0.53 *	*1.68 *
		**32.38 ± 0.61**	**1.89**	**35.12 ± 0.10**	**0.28**	**30.46 ± 0.92**	**3.03**	**31.05 ± 0.38**	**1.23**	**31.35 ± 0.70**	**2.22**
10^1^	5.0 10^0^	2/3^**^		1/3^**^		2/3^**^		*31.52 ± 1.33 *	*4.20 *	1/3^**^	
		1/3^**^		1/3^**^		**32.76 ± 0.70**	**2.12**	1/3^**^		2/3^**^	
1	0.5	1/3^**^		0/3^**^		0/3^**^		0/3^**^		0/3^**^	
		0/3^**^		0/3^**^		0/3^**^		0/3^**^		2/3^**^	

Efficiency		*99% *		*99% *		*100% *		*101% *		*90% *	
		**96%**		**107%**		**97%**		**93%**		**102%**	

*R* ^2^		*0.999 *		*0.997 *		*0.997 *		*0.999 *		*0.999 *	
		**0.999**		**0.991**		**0.997**		**0.994**		**0.998**	

*y*-intercept		*36.65 *		*39.55 *		*36.31 *		*34.73 *		*37.67 *	
		**39.38**		**41.83**		**38.01**		**38.50**		**38.01**	

^*^CV (%) = standard deviation/mean Ct × 100.

CV was not calculated when some of the parallels failed to amplify.

^**^ *X*/*X*: number of positives/number of experiments.

^***^Average Ct values: mean ± standard deviation.

^****^All PCR comparisons were run on the Rotor Gene Q MDx thermocycler (in italic font) and on the Mx3005P thermocycler (in bold).

**Table 3:** Comparison of qRT-PCR SYBR Green (one-step) kits. Results of five different qRT-PCR kits on a range of seven triplicate dilutions of EBLV-1 *in vitro* RNA transcripts.

Testing of different qRT-PCR kits (one-step)											
Number of copies/sample	Number of copies/rxn	Quantitect-1 step		Rotor Gene-1 step		Verso-1 step		SSIII Platinium-1 step		qScript-1 step	
		Average Ct^***^	CV (%)^*^	Average Ct^***^	CV (%)^*^	Average Ct^***^	CV (%)^*^	Average Ct^***^	CV (%)^*^	Average Ct^***^	CV (%)^*^
10^6^	2.0 10^6^	*16.66 ± 0.08 *	*0.45 *	*17.15 ± 0.05 *	*0.29 *	*16.99 ± 0.06 *	*0.38 *	*14.52 ± 0.19 *	*0.32 *	*13.47 ± 0.03 *	*0.19 *
		**17.79 ± 0.24**	**1.32**	**15.88 ± 0.22**	**1.36**	**18.34 ± 0.32**	**1.72**	**16.22 ± 0.04**	**0.22**	**18.48 ± 0.04**	**0.20**
10^5^	2.0 10^5^	*20.06 ± 0.10 *	*0.52 *	*20.62 ± 0.04 *	*0.17 *	*20.40 ± 0.11 *	*0.52 *	*17.90 ± 0.21 *	*1.18 *	*16.55 ± 0.03 *	*0.19 *
		**21.13 ± 0.45**	**2.12**	**19.42 ± 0.20**	**1.04**	**21.60 ± 0.15**	**0.71**	**19.64 ± 0.41**	**2.11**	**22.38 ± 0.15**	**0.68**
10^4^	2.0 10^4^	*23.58 ± 0.14 *	*0.58 *	*23.91 ± 0.08 *	*0.33 *	*23.82 ± 0.20 *	*0.83 *	*21.43 ± 0.12 *	*0.57 *	*19.86 ± 0.04 *	*0.18 *
		**24.60 ± 0.20**	**0.83**	**22.87 ± 0.16**	**0.69**	**25.17 ± 0.17**	**0.69**	**23.08 ± 0.27**	**1.16**	**26.11 ± 0.10**	**0.38**
10^3^	2.0 10^3^	*26.70 ± 0.24 *	*0.91 *	*27.45 ± 0.28 *	*1.01 *	*26.93 ± 0.03 *	*0.11 *	*24.90 ± 0.23 *	*0.92 *	*23.13 ± 0.16 *	*0.67 *
		**27.95 ± 0.26**	**0.93**	**26.34 ± 0.33**	**1.24**	**28.63 ± 0.30**	**1.06**	**26.14 ± 0.10**	**0.38**	**29.54 ± 0.25**	**0.86**
10^2^	2.0 10^2^	*30.42 ± 0.08 *	*0.26 *	2/3^**^		*30.19 ± 0.60 *	*1.98 *	0/3^**^		0/3^**^	
		**31.98 ± 0.17**	**0.53**	**29.77 ± 0.39**	**1.32**	**31.74 ± 0.79**	**2.50**	**28.93 ± 0.08**	**0.28**	**31.07 ± 0.37**	**1.18**
10^1^	2.0 10^1^	*33.24 ± 0.29 *	*0.87 *	1/3^**^		*33.27 ± 1.53 *	*4.60 *	0/3^**^		0/3^**^	
		**34.34 ± 1.03**	**2.99**	**32.84 ± 0.44**	**1.33**	2/3^**^		**29.91 ± 0.10**	**0.33**	0/3^**^	
1	2	0/3^**^		1/3^**^		0/3^**^		0/3^**^		0/3^**^	
		0/3^**^		0/3^**^		2/3^**^		0/3^**^		0/3^**^	

Efficiency		*98% *		*96% *		*100% *		*94% *		*104% *	
		**97%**		**94%**		**95%**		**100%**		**87%**	

*R* ^2^		*0.998 *		*0.999 *		*0.999 *		*0.998 *		*0.999 *	
		**0.996**		**0.998**		**0.997**		**0.996**		**0.998**	

*y*-intercept		*36.89 *		*37.67 *		*36.99 *		*35.29 *		*32.78 *	
		**38.15**		**36.80**		**38.93**		**38.93**		**40.74**	

^*^CV (%) = standard deviation/mean Ct × 100.

CV was not calculated when some of the parallels failed to amplify.

^**^ *X*/*X*: number of positives/number of experiments.

^***^Average Ct values: mean ± standard deviation.

^****^All PCR comparisons were run on the Rotor Gene Q MDx thermocycler (in italic font) and on the Mx3005P thermocycler (in bold).

**Table 4:** Detection limit of PCR (LOD 95%) for two-step and one-step SYBR Green RT-PCR.

Two-step RT-qPCR									
IScript RT + qPCR SsoFast BioRad		QuantiTect RT + qPCR Quantitect QIAGEN		Maxima RT + qPCR Maxima FERMENTAS		SS Vilo RT + qPCR Express INVITROGEN		qScript RT + qPCR Perfecta QUANTA	
Number of copies/sample	Number of copies/rxn	Number of copies/sample	Number of copies/rxn	Number of copies/sample	Number of copies/rxn	Number of copies/sample	Number of copies/rxn	Number of copies/sample	Number of copies/rxn
*110 *	*55 *	*171 *	*86 *	*78 *	*39 *	*41 *	*21 *	*88 *	*44 *
**88**	**44**	**160**	**80**	**76**	**38**	**45**	**22**	**67**	**34**

One-step qRT-PCR									
Quantitect-1 step QIAGEN		Rotor Gene-1 step QIAGEN		Verso-1 step FERMENTAS		SSIII Platinium-1 step INVITROGEN		qScript-1 step QUANTA	
Number of copies/sample	Number of copies/rxn	Number of copies/sample	Number of copies/rxn	Number of copies/sample	Number of copies/rxn	Number of copies/sample	Number of copies/rxn	Number of copies/sample	Number of copies/rxn

*20 *	*40 *	*497 *	*994 *	*127 *	*254 *	*321 *	*641 *	*406 *	*812 *
**22**	**44**	**15**	**30**	**101**	**202**	**50**	**100**	**981**	**1962**

^*^All LOD 95% results were carried out using Probit model.

^*^All PCR comparisons were run on the Rotor Gene Q MDx thermocycler (in italic font) and on the Mx3005P thermocycler (in bold).

**Table:** (a) 2-step methods

Kit	Number of copies/sample	Intra-assay variability^***^											Inter-assay variability^***^	
		Run 1			Run 2			Run 3			Average			
		Average Ct ± SD Ct^*^	CV (%)^**^	Number of pos.	Average Ct ± SD Ct^*^	CV (%)^**^	Number of pos.	Average Ct ± SD Ct^*^	CV (%)^**^	Number of pos.	Ct ± SD Ct^*^	CV (%)^**^	Average Ct ± SD Ct	CV (%)^**^
IScript RT + qPCR SsoFast	*100 *	*30.46 ± 0.41 *	*1.35 *	*8 *	*30.08 ± 0.57 *	*1.90 *	*8 *	*29.82 ± 0.41 *	*1.38 *	*8 *	*30.12 ± 0.46 *	*1.54 *	*30.12 ± 0.52 *	*1.74 *
	*50 *	*31.11 ± 0.93 *	*2.99 *	*6 *	*31.84 ± 1.01 *	*3.19 *	*6 *	*30.80 ± 0.46 *	*1.51 *	*8 *	*31.25 ± 0.80 *	*2.56 *	*31.20 ± 0.88 *	*2.83 *
	*25 *	*32.49 ± 0.59 *	*1.83 *	*6 *	*31.60 ± 0.67 *	*2.12 *	*8 *	*31.27 ± 0.68 *	*2.19 *	*6 *	*31.79 ± 0.65 *	*2.04 *	*31.77 ± 0.80 *	*2.51 *
	*10 *	*32.38 ± 0.91 *	*2.81 *	*6 *	*32.69 ± 1.19 *	*3.63 *	*6 *	*31.97 ± 0.76 *	*2.39 *	*5 *	*32.35 ± 0.95 *	*2.94 *	*32.37 ± 0.97 *	*2.99 *
	*5 *	*33.14 *	*— *	*1 *	*— *	*— *	*0 *	*31.98 ± 0.05 *	*0.15 *	*3 *	*32.56 ± 0.05 *	*0.15 *	*32.27 ± 0.58 *	*1.81 *
	*0.5 *	*32.17 *	*— *	*1 *	*— *	*— *	*0 *	*32.11 ± 0.76 *	*2.36 *	*3 *	*32.14 ± 0.76 *	*2.36 *	*32.13 ± 0.62 *	*1.93 *
	**250**	**30.71 ± 0.49**	**1.61**	**8**	**30.70 ± 0.39**	**1.28**	**8**	**30.61 ± 0.33**	**1.08**	**8**	**30.67 ± 0.41**	**1.32**	**30.67 ± 0.40**	**1.29**
	**100**	**32.30 ± 0.74**	**2.28**	**8**	**32.03 ± 0.90**	**2.81**	**8**	**32.19 ± 0.40**	**1.24**	**8**	**32.17 ± 0.68**	**2.11**	**32.17 ± 0.69**	**2.14**
	**50**	**34.02 ± 0.93**	**2.73**	**6**	**33.24 ± 0.48**	**1.44**	**8**	**33.16 ± 0.93**	**2.87**	**8**	**33.47 ± 0.79**	**2.35**	**33.42 ± 0.85**	**2.55**
	**25**	**34.63 ± 1.02**	**2.95**	**8**	**33.78 ± 0.75**	**2.23**	**6**	**34.24 ± 0.88**	**2.58**	**6**	**34.21 ± 0.89**	**2.59**	**34.25 ± 0.93**	**2.72**
	**10**	**35.95 ± 1.12**	**3.12**	**5**	**35.26 ± 0.47**	**1.34**	**5**	**34.68 ± 00.63**	**1.80**	**6**	**35.30 ± 0.74**	**2.09**	**35.26 ± 0.90**	**2.56**
	**5**	**33.84 ± 0.62**	**1.84**	**3**	**—**	**—**	**0**	**—**	**—**	**0**	**34.83 ± 0.60**	**1.71**	**33.84 ± 0.62**	**1.84**

QuantiTect RT + qPCR Quantitect	*250 *	*31.37 ± 0.69 *	*2.20 *	*8 *	*31.45 ± 0.52 *	*1.65 *	*8 *	*31.22 ± 0.46 *	*1.47 *	*8 *	*31.35 ± 0.56 *	*1.77 *	*31.35 ± 0.55 *	*1.75 *
	*100 *	*32.46 ± 0.80 *	*2.46 *	*7 *	*32.72 ± 0.92 *	*2.81 *	*7 *	*33.90 ± 0.90 *	*2.65 *	*7 *	*33.03 ± 0.87 *	*2.64 *	*33.03 ± 1.05 *	*3.18 *
	*50 *	*33.59 ± 0.60 *	*1.79 *	*4 *	*34.34 ± 2.32 *	*6.75 *	*5 *	*33.66 ± 0.79 *	*2.35 *	*6 *	*33.86 ± 1.24 *	*3.63 *	*33.87 ± 1.40 *	*4.13 *
	*25 *	*32.98 ± 0.74 *	*2.24 *	*4 *	*33.58 ± 1.18 *	*3.51 *	*5 *	*34.12 ± 2.07 *	*6.08 *	*4 *	*33.56 ± 1.33 *	*3.94 *	*33.56 ± 1.38 *	*4.10 *
	*10 *	*33.54 ± 0.18 *	*0.54 *	*3 *	*33.86 ± 0.61 *	*1.80 *	*2 *	*34.73 ± 0.62 *	*1.79 *	*2 *	*34.04 ± 0.47 *	*1.38 *	*33.97 ± 0.65 *	*1.92 *
	*5 *	*— *	*— *	*0 *	*35.52 *	*— *	*1 *	*— *	*— *	*0 *	*35.52 *	*— *	*35.52 *	*— *
	**500**	**34.09 ± 0.36**	**1.35**	**8**	**36.37 ± 1.18**	**3.26**	**6**	**32.97 ± 0.38**	**1.16**	**8**	**34.30 ± 1.54**	**1.92**	**34.30 ± 1.54**	**4.48**
	**250**	**36.42 ± 0.46**	**6.43**	**8**	**33.66 ± 0.80**	**2.39**	**8**	**33.61 ± 0.83**	**2.46**	**8**	**34.56 ± 1.97**	**3.76**	**34.56 ± 1.97**	**5.69**
	**100**	**34.39 ± 2.34**	**2.57**	**8**	**34.60 ± 0.98**	**2.84**	**8**	**34.50 ± 0.91**	**2.65**	**8**	**34.50 ± 0.89**	**2.69**	**34.50 ± 0.89**	**2.58**
	**50**	**36.28 ± 0.88**	**4.75**	**6**	**35.07 ± 0.91**	**2.59**	**7**	**34.92 ± 1.04**	**2.97**	**7**	**35.38 ± 1.32**	**3.44**	**35.38 ± 1.32**	**3.74**
	**25**	**35.97 ± 1.72**	**2.30**	**5**	**36.28 ± 0.88**	**2.42**	**4**	**36.44 ± 0.78**	**2.14**	**5**	**36.23 ± 0.79**	**2.29**	**36.23 ± 0.79**	**2.17**
	**10**	**—**	**—**	**0**	**—**	**—**	**0**	**37.65 ± 0.79**	**2.10**	**2**	**37.65 ± 0.79**	**2.10**	**37.65 ± 0.79**	**2.10**

Maxima RT + qPCR Maxima	*100 *	*31.83 ± 0.84 *	*2.63 *		*31.38 ± 0.22 *	*0.70 *		*30.09 ± 0.41 *	*1.37 *		*31.1 ± 0.49 *	*1.57 *	*31.1 ± 0.92 *	*2.95 *
	*50 *	*33.35 ± 1.09 *	*3.26 *	*8 *	*33.42 ± 0.88 *	*2.64 *	*5 *	*31.12 ± 0.64 *	*2.05 *	*8 *	*32.63 ± 0.87 *	*2.65 *	*32.51 ± 1.40 *	*4.32 *
	*25 *	*33.67 ± 0.88 *	*2.61 *	*5 *	*32.77 ± 1.12 *	*3.40 *	*6 *	*32.47 ± 0.72 *	*2.21 *	*7 *	*32.97 ± 0.90 *	*2.74 *	*32.90 ± 0.99 *	*3.02 *
	*10 *	*37.16 ± 4.64 *	*12.49 *	*3 *	*34.00 ± 1.41 *	*4.16 *	*4 *	*34.35 ± 1.33 *	*3.88 *	*5 *	*35.17 ± 2.46 *	*6.84 *	*34.94 ± 2.63 *	*7.54 *
	*5 *	*35.44 ± 0.24 *	*0.68 *	*2 *	*38.65 *	*— *	*1 *	*34.58 ± 1.24 *	*3.57 *	*3 *	*36.22 ± 0.74 *	*2.12 *	*35.55 ± 1.76 *	*4.96 *
	*2.5 *	*— *	*— *	*0 *	*33.14 *	*— *	*1 *	*33.47 ± 1.76 *	*5.26 *	*2 *	*33.30 ± 1.76 *	*5.26 *	*33.36 ± 1.26 *	*3.77 *
	**250**	**29.28 ± 0.58**	**1.99**	**8**	**29.05 ± 0.38**	**1.30**	**8**	**29.04 ± 0.39**	**1.35**	**8**	**29.12 ± 0.45**	**1.55**	**29.12 ± 0.45**	**1.56**
	**100**	**30.53 ± 0.56**	**1.85**	**8**	**30.17 ± 0.48**	**1.59**	**8**	**30.31 ± 0.43**	**1.43**	**8**	**30.34 ± 0.50**	**1.62**	**30.34 ± 0.50**	**1.64**
	**50**	**31.54 ± 1.25**	**3.97**	**8**	**31.88 ± 1.37**	**4.29**	**8**	**31.72 ± 0.56**	**1.77**	**8**	**31.71 ± 1.08**	**3.34**	**31.71 ± 1.08**	**3.40**
	**25**	**33.00 ± 1.15**	**3.48**	**6**	**32.38 ± 0.79**	**2.43**	**7**	**32.38 ± 0.82**	**2.54**	**7**	**32.56 ± 0.92**	**2.82**	**32.56 ± 0.92**	**2.81**
	**10**	**33.08 ± 0.64**	**1.93**	**3**	**33.02 ± 1.38**	**4.17**	**4**	**33.40 ± 1.46**	**4.38**	**4**	**33.17 ± 1.15**	**3.49**	**33.17 ± 1.15**	**3.47**
	**50**	**33.03 ± 0.15**	**0.45**	**2**	**33.55**	**—**	**1**	**32.99**	**—**	**1**	**33.15 ± 0.28**	**0.45**	**33.15 ± 0.28**	**0.85**

qScript RT + qPCR Perfecta	*250 *	*27.04 ± 0.24 *	*0.88 *	*8 *	*26.75 ± 0.26 *	*0.98 *	*8 *	*26.77 ± 0.24 *	*0.90 *	*8 *	*26.85 ± 0.25 *	*0.92 *	*26.85 ± 0.27 *	*1.01 *
	*100 *	*30.54 ± 0.54 *	*1.76 *	*8 *	*29.84 ± 0.54 *	*1.82 *	*8 *	*30.46 ± 1.07 *	*3.50 *	*8 *	*30.28 ± 0.72 *	*2.36 *	*30.28 ± 0.79 *	*2.61 *
	*50 *	*32.03 ± 2.90 *	*9.05 *	*7 *	*30.72 ± 0.89 *	*2.92 *	*8 *	*30.78 ± 0.82 *	*2.67 *	*8 *	*31.18 ± 1.54 *	*4.88 *	*31.14 ± 1.77 *	*5.68 *
	*25 *	*32.50 ± 0.98 *	*3.02 *	*7 *	*32.41 ± 1.33 *	*4.10 *	*7 *	*33.63 ± 2.16 *	*6.42 *	*5 *	*32.85 ± 1.49 *	*4.52 *	*32.76 ± 1.49 *	*4.56 *
	*10 *	*32.22 ± 0.81 *	*2.52 *	*5 *	*32.87 ± 0.46 *	*1.39 *	*3 *	*32.70 ± 0.56 *	*1.71 *	*2 *	*32.60 ± 0.61 *	*1.87 *	*32.51 ± 0.69 *	*2.11 *
	*5 *	*32.78 ± 0.15 *	*0.45 *	*2 *	*— *	*— *	*0 *	*33.56 ± 1.55 *	*4.61 *	*2 *	*33.18 ± 0.85 *	*2.53 *	*33.18 ± 1.00 *	*3.03 *
	**250**	**29.47 ± 0.22**	**0.74**	**8**	**29.49 ± 0.54**	**1.83**	**7**	**29.82 ± 0.38**	**1.29**	**8**	**29.60 ± 0.41**	**1.29**	**29.60 ± 0.41**	**1.39**
	**100**	**30.99 ± 1.09**	**3.52**	**8**	**31.14 ± 1.26**	**4.04**	**8**	**31.02 ± 0.52**	**1.69**	**8**	**31.05 ± 0.96**	**3.08**	**31.05 ± 0.96**	**3.11**
	**50**	**31.87 ± 0.60**	**1.88**	**8**	**31.37 ± 0.57**	**1.80**	**8**	**33.13 ± 1.12**	**3.37**	**8**	**32.12 ± 1.08**	**2.35**	**32.12 ± 1.08**	**3.35**
	**25**	**33.35 ± 2.99**	**8.96**	**7**	**32.49 ± 0.68**	**2.11**	**8**	**32.71 ± 0.87**	**2.65**	**8**	**32.83 ± 1.72**	**4.57**	**32.83 ± 1.72**	**5.24**
	**10**	**33.00 ± 0.49**	**1.47**	**5**	**33.45 ± 0.18**	**0.53**	**2**	**32.98 ± 0.74**	**2.25**	**4**	**33.07 ± 0.54**	**1.42**	**33.07 ± 0.54**	**1.65**
	**5**	**33.04 ± 1.01**	**3.06**	**2**	**33.03 ± 0.74**	**2.23**	**2**	**33.59**	**—**	**1**	**33.14 ± 0.67**	**2.65**	**33.14 ± 0.67**	**2.03**

SS Vilo RT + qPCR Express	*100 *	*27.68 ± 0.59 *	*2.13 *	*8 *	*27.82 ± 0.55 *	*1.97 *	*8 *	*27.59 ± 0.29 *	*1.06 *	*8 *	*27.70 ± 0.48 *	*1.72 *	*27.70 ± 0.48 *	*1.74 *
	*50 *	*29.42 ± 0.62 *	*2.11 *	*8 *	*29.27 ± 0.54 *	*1.85 *	*8 *	*29.21 ± 0.60 *	*2.06 *	*8 *	*29.30 ± 0.59 *	*2.01 *	*29.30 ± 0.57 *	*1.95 *
	*25 *	*30.38 ± 0.67 *	*2.21 *	*6 *	*30.22 ± 1.24 *	*4.11 *	*8 *	*29.60 ± 0.48 *	*1.62 *	*8 *	*30.07 ± 0.80 *	*2.64 *	*30.04 ± 0.90 *	*3.00 *
	*10 *	*31.36 ± 0.49 *	*1.58 *	*7 *	*31.30 ± 0.71 *	*2.27 *	*7 *	*31.92 ± 0.78 *	*2.45 *	*6 *	*31.53 ± 0.66 *	*2.10 *	*31.51 ± 0.69 *	*2.19 *
	*5 *	*31.49 ± 0.53 *	*1.68 *	*4 *	*31.63 ± 0.98 *	*3.08 *	*4 *	*31.64 *	*— *	*1 *	*31.59 ± 0.75 *	*2.38 *	*31.57 ± 0.68 *	*2.16 *
	*0.5 *	*— *	*— *	*0 *	*— *	*— *	*0 *	*31.59 *	*— *	*1 *	*31.59 *	*— *	*31.59 *	*— *
	**100**	**31.78 ± 0.53**	**1.67**	**8**	**30.13 ± 0.32**	**1.07**	**8**	**30.75 ± 0.65**	**2.11**	**8**	**30.89 ± 0.85**	**1.62**	**30.89 ± 0.85**	**2.76**
	**50**	**32.96 ± 0.42**	**1.29**	**8**	**31.34 ± 0.50**	**1.61**	**8**	**31.49 ± 0.97**	**3.07**	**8**	**31.93 ± 0.99**	**1.99**	**31.93 ± 0.99**	**3.09**
	**25**	**33.72 ± 1.05**	**3.10**	**7**	**32.15 ± 0.98**	**3.06**	**8**	**32.09 ± 0.22**	**0.69**	**6**	**32.66 ± 1.13**	**2.28**	**32.66 ± 1.13**	**3.46**
	**10**	**34.44 ± 0.60**	**1.74**	**7**	**33.61 ± 0.46**	**1.35**	**5**	**33.30 ± 0.43**	**1.29**	**6**	**33.83 ± 0.71**	**1.46**	**33.83 ± 0.71**	**2.09**
	**5**	**35.21 ± 0.53**	**1.51**	**7**	**32.64 ± 0.36**	**1.11**	**5**	**32.99 ± 0.06**	**0.17**	**2**	**33.97 ± 1.35**	**0.93**	**33.97 ± 1.35**	**3.98**
	**2.5**	**39.56 ± 1.86**	**4.71**	**3**	**34.35**		**1**	**33.55 ± 0.57**	**1.69**	**3**	**36.24 ± 3.32**	**3.20**	**36.24 ± 3.32**	**9.16**

The intra-  and interassay variability and coefficient of variations were calculated by the formulas: inter-assay CV = standard deviation of all replicates/(mean Ct-value of all replicates) × 100; intra-assay CV = mean of the CVs of all runs.

^*^Average Ct values: mean ± standard deviation.

^**^CV (%) = standard deviation/mean Ct × 100.

^***^All PCR comparisons were run on the Rotor Gene Q MDx thermocycler (in italic font) and on the Mx3005P thermocycler (in bold).

**Table:** (b) 1 step methods

Kit	Number of copies/sample	Intra-assay variability^***^											Inter-assay variability^***^	
		Run 1			Run 2			Run 3			Average			
		Average Ct ± SD Ct^*^	CV^**^	Number of pos.	Average Ct ± SD Ct^*^	CV^**^	Number of pos.	Average Ct ± SD Ct^*^	CV^**^	Number of pos.	Ct ± SD Ct^*^	CV^**^	Average Ct ± SD Ct^*^	CV^**^
Quantitect 1 step	*50 *	*29.76 ± 0.64 *	*2.16 *	*8 *	*28.94 ± 0.55 *	*1.89 *	*8 *	*29.27 ± 0.23 *	*0.79 *	*8 *	*29.32 ± 0.47 *	*1.61 *	*29.32 ± 0.59 *	*1.06 *
	*25 *	*30.90 ± 0.88 *	*2.86 *	*8 *	*29.84 ± 1.09 *	*3.65 *	*8 *	*30.98 ± 0.76 *	*2.45 *	*8 *	*30.57 ± 0.91 *	*2.99 *	*30.57 ± 1.03 *	*2.02 *
	*10 *	*31.72 ± 1.04 *	*3.26 *	*7 *	*32.05 ± 1.06 *	*3.32 *	*7 *	*32.30 ± 0.61 *	*1.88 *	*8 *	*32.03 ± 0.90 *	*2.82 *	*32.04 ± 0.90 *	*3.36 *
	*5 *	*33.34 ± 1.20 *	*3.60 *	*6 *	*32.38 ± 0.74 *	*2.28 *	*7 *	*32.85 ± 0.84 *	*2.55 *	*7 *	*32.86 ± 0.92 *	*2.81 *	*32.83 ± 0.96 *	*2.81 *
	*2.5 *	*32.51 ± 0.60 *	*1.85 *	*4 *	*33.90 ± 0.33 *	*0.96 *	*4 *	*34.45 ± 1.52 *	*4.42 *	*5 *	*33.62 ± 0.82 *	*2.41 *	*33.68 ± 1.27 *	*3.77 *
	*1 *	*33.38 ± 0.96 *	*2.88 *	*4 *	*32.03 ± 0.26 *	*0.80 *	*3 *	*32.92 ± 0.47 *	*1.43 *	*5 *	*32.77 ± 0.56 *	*1.70 *	*32.85 ± 0.79 *	*2.42 *
	**50**	**30.92 ± 0.47**	**1.52**	**8**	**31.23 ± 1.29**	**0.40**	**8**	**31.11 ± 1.32**	**0.41**	**8**	**31.08 ± 1.38**	**0.43**	**31.08 ± 0.43**	**1.38**
	**25**	**31.77 ± 0.73**	**2.29**	**8**	**31.92 ± 1.49**	**0.48**	**8**	**31.91 ± 0.93**	**0.30**	**8**	**31.87 ± 1.57**	**0.50**	**31.87 ± 0.51**	**1.61**
	**10**	**32.60 ± 0.47**	**1.44**	**8**	**32.81 ± 0.61**	**1.87**	**5**	**32.49 ± 0.65**	**1.99**	**8**	**32.63 ± 0.58**	**1.77**	**32.63 ± 0.57**	**1.75**
	**5**	**33.69 ± 0.85**	**2.53**	**7**	**33.82 ± 2.91**	**0.98**	**5**	**33.28 ± 2.50**	**0.83**	**6**	**33.60 ± 2.65**	**0.89**	**33.57 ± 0.86**	**2.57**
	**2.5**	**34.36 ± 0.73**	**2.14**	**5**	**33.65 ± 1.88**	**0.63**	**2**	**33.27 ± 1.88**	**0.62**	**3**	**33.76 ± 1.96**	**0.66**	**33.80 ± 0.80**	**2.36**
	**1**	**34.55 ± 1.77**	**5.13**	**3**	**34.97 ± 1.17**	**0.41**	**5**	**33.92 ± 3.41**	**1.16**	**1**	**34.48 ± 3.24**	**1.11**	**34.45 ± 1.37**	**3.97**

Rotor Gene 1 step	*1000 *	*26.40 ± 0.45 *	*1.69 *	*8 *	*23.74 ± 0.40 *	*1.67 *	*8 *	*27.01 ± 1.18 *	*4.38 *	*8 *	*25.72 ± 1.62 *	*2.58 *	*25.72 ± 1.62 *	*6.32 *
	*500 *	*27.49 ± 0.46 *	*1.66 *	*8 *	*24.29 ± 0.34 *	*1.39 *	*8 *	*27.42 ± 1.04 *	*3.78 *	*8 *	*26.40 ± 1.66 *	*2.28 *	*26.40 ± 1.66 *	*6.28 *
	*250 *	*28.73 ± 0.76 *	*2.65 *	*8 *	*24.90 ± 0.49 *	*1.96 *	*8 *	*29.27 ± 0.60 *	*2.04 *	*7 *	*27.56 ± 2.09 *	*2.22 *	*27.56 ± 2.09 *	*7.58 *
	*100 *	*30.70 ± 0.21 *	*0.67 *	*3 *	*25.50 *	*— *	*1 *	*31.19 ± 0.66 *	*2.11 *	*2 *	*30.00 ± 2.24 *	*1.39 *	*30.00 ± 2.24 *	*7.46 *
	*50 *	*30.31 *	*— *	*1 *	*— *	*— *	*0 *	*— *	*— *	*0 *	*30.31 *	*— *	*30.31 *	*— *
	*25 *	*31.12 ± 0.50 *	*1.61 *	*2 *	*— *	*— *	*0 *	*— *	*— *	*0 *	*32.12 ± 0.50 *	*1.61 *	*32.12 ± 0.50 *	*1.61 *
	**50**	**28.87 ± 0.99**	**3.41**	**8**	**29.25 ± 0.74**	**2.52**	**8**	**27.97 ± 0.65**	**2.33**	**8**	**28.70 ± 0.79**	**2.76**	**28.70 ± 0.94**	**3.29**
	**25**	**29.49 ± 1.01**	**3.44**	**8**	**30.36 ± 0.70**	**2.29**	**8**	**28.57 ± 0.39**	**1.36**	**8**	**29.47 ± 0.70**	**2.36**	**29.47 ± 1.03**	**3.50**
	**10**	**30.29 ± 1.20**	**3.98**	**8**	**30.61 ± 1.44**	**4.70**	**8**	**28.76 ± 0.40**	**1.39**	**8**	**29.89 ± 1.01**	**3.36**	**29.89 ± 1.34**	**4.50**
	**5**	**30.82 ± 1.06**	**3.43**	**6**	**31.47 ± 1.30**	**4.12**	**6**	**29.90 ± 0.40**	**1.35**	**8**	**30.73 ± 0.92**	**2.97**	**30.65 ± 1.12**	**3.66**
	**2.5**	**30.87 ± 1.14**	**3.68**	**6**	**31.69 ± 0.57**	**1.81**	**5**	**29.10 ± 0.37**	**1.26**	**5**	**30.55 ± 0.69**	**2.25**	**30.57 ± 1.31**	**4.29**
	**1**	**31.22**	**—**	**1**	**31.53**	**—**	**1**	**32.01 ± 0.44**	**1.37**	**5**	**31.59 ± 0.44**	**1.37**	**31.83 ± 0.48**	**1.51**

Verso 1 step	*500 *	*26.50 ± 0.18 *	*0.68 *	*8 *	*26.56 ± 0.14 *	*0.53 *	*8 *	*26.79 ± 0.23 *	*0.85 *	*8 *	*26.61 ± 0.18 *	*0.69 *	*26.61 ± 0.22 *	*0.82 *
	*250 *	*27.43 ± 0.18 *	*0.65 *	*8 *	*27.96 ± 0.27 *	*0.98 *	*8 *	*27.89 ± 0.36 *	*1.27 *	*8 *	*27.76 ± 0.27 *	*0.97 *	*27.76 ± 0.36 *	*1.30 *
	*100 *	*28.94 ± 0.50 *	*1.72 *	*8 *	*29.42 ± 0.53 *	*1.80 *	*8 *	*29.91 ± 1.18 *	*3.96 *	*8 *	*29.43 ± 0.74 *	*2.49 *	*29.43 ± 0.87 *	*2.95 *
	*50 *	*30.49 ± 0.44 *	*1.43 *	*7 *	*31.38 ± 0.86 *	*2.74 *	*8 *	*31.29 ± 1.52 *	*4.87 *	*8 *	*31.05 ± 0.94 *	*3.01 *	*31.08 ± 1.09 *	*3.50 *
	*25 *	*32.82 ± 1.14 *	*3.46 *	*7 *	*32.90 ± 0.52 *	*1.57 *	*5 *	*32.50 ± 0.92 *	*2.82 *	*7 *	*32.74 ± 0.86 *	*2.62 *	*32.72 ± 0.90 *	*2.74 *
	*10 *	*33.42 ± 0.97 *	*2.91 *	*3 *	*35.22 ± 1.05 *	*2.99 *	*2 *	*34.42 *	*— *	*1 *	*34.35 ± 1.01 *	*2.95 *	*34.19 ± 1.18 *	*3.44 *
	**250**	**29.39 ± 0.48**	**1.64**	**8**	**29.40 ± 0.23**	**0.79**	**8**	**29.10 ± 0.53**	**1.83**	**8**	**29.20 ± 0.42**	**1.42**	**29.30 ± 0.44**	**1.50**
	**100**	**30.55 ± 0.25**	**0.80**	**8**	**30.32 ± 0.38**	**1.25**	**8**	**30.02 ± 0.24**	**0.82**	**6**	**30.29 ± 0.29**	**0.96**	**30.32 ± 0.36**	**1.19**
	**50**	**31.16 ± 0.50**	**1.59**	**8**	**31.10 ± 0.52**	**1.68**	**8**	**30.87 ± 0.42**	**1.35**	**7**	**31.04 ± 0.48**	**1.54**	**31.05 ± 0.48**	**1.53**
	**25**	**31.71 ± 0.32**	**1.02**	**7**	**31.61 ± 0.42**	**1.34**	**8**	**31.35 ± 0.58**	**1.84**	**8**	**31.56 ± 0.44**	**1.40**	**31.55 ± 0.46**	**1.47**
	**10**	**31.86 ± 0.54**	**1.69**	**7**	**31.76 ± 0.29**	**0.91**	**7**	**31.65 ± 0.64**	**2.01**	**4**	**31.76 ± 0.49**	**1.54**	**31.77 ± 0.46**	**1.45**
	**5**	**33.09 ± 0.98**	**2.97**	**5**	**33.13**		**1**	**33.43 ± 0.74**	**2.23**	**4**	**33.22 ± 0.86**	**2.60**	**33.26 ± 0.80**	**2.39**

qScript 1 step	*1000 *	*23.24 ± 0.12 *	*0.53 *	*8 *	*22.97 ± 0.24 *	*1.03 *	*8 *	*23.21 ± 0.09 *	*0.37 *	*8 *	*23.14 ± 0.15 *	*0.64 *	*23.14 ± 0.20 *	*0.86 *
	*750 *	*23.64 ± 0.21 *	*0.88 *	*8 *	*23.29 ± 0.08 *	*0.35 *	*8 *	*23.52 ± 0.28 *	*1.21 *	*8 *	*23.48 ± 0.19 *	*0.81 *	*23.48 ± 0.25 *	*1.06 *
	*500 *	*24.46 ± 0.32 *	*1.32 *	*8 *	*24.06 ± 0.24 *	*0.98 *	*8 *	*24.07 ± 0.29 *	*1.22 *	*8 *	*24.19 ± 0.28 *	*1.17 *	*24.19 ± 0.33 *	*1.37 *
	*250 *	*25.38 ± 0.27 *	*1.07 *	*8 *	*24.93 ± 0.26 *	*1.05 *	*8 *	*25.14 ± 0.34 *	*1.37 *	*8 *	*25.15 ± 0.29 *	*1.16 *	*25.15 ± 0.34 *	*1.35 *
	*100 *	*26.26 ± 0.31 *	*1.16 *	*8 *	*25.79 ± 0.24 *	*0.93 *	*7 *	*25.95 ± 0.47 *	*1.80 *	*5 *	*26.00 ± 0.34 *	*1.30 *	*26.02 ± 0.38 *	*1.46 *
	*50 *	*— *	*— *	*0 *	*— *	*— *	*0 *	*26.45 ± 0.18 *	*0.70 *	*2 *	*26.45 ± 0.18 *	*0.70 *	*26.45 ± 0.18 *	*0.70 *
	**2500**	**28.13 ± 0.83**	**2.94**	**8**	**27.78 ± 0.45**	**1.60**	**8**	**26.32 ± 0.30**	**1.13**	**8**	**27.41 ± 0.52**	**1.89**	**27.41 ± 0.97**	**3.53**
	**1000**	**28.78 ± 0.09**	**0.33**	**8**	**29.40 ± 1.58**	**5.39**	**8**	**24.89 ± 0.30**	**1.22**	**8**	**27.69 ± 0.66**	**2.31**	**27.69 ± 2.23**	**8.05**
	**750**	**29.08 ± 0.24**	**0.82**	**8**	**28.92 ± 0.25**	**0.85**	**8**	**28.75 ± 0.11**	**0.38**	**8**	**28.92 ± 0.20**	**0.68**	**28.92 ± 0.24**	**0.84**
	**500**	**30.69 ± 0.48**	**1.57**	**7**	**29.76 ± 0.55**	**1.86**	**8**	**27.63 ± 0.56**	**2.01**	**5**	**29.36 ± 0.53**	**1.81**	**29.66 ± 1.26**	**4.24**
	**250**	**31.22 ± 0.23**	**0.73**	**7**	**30.05 ± 0.37**	**1.24**	**8**	**—**	**—**	**0**	**30.64 ± 0.30**	**0.99**	**30.60 ± 0.67**	**2.20**
	**100**	**—**	**—**	**0**	**30.00**	**—**	**1**	**—**	**—**	**0**	**30.00**	**—**	**30.00**	**—**

SSIII Platinium 1 step	*1000 *	*24.79 ± 0.24 *	*0.97 *	*8 *	*23.81 ± 0.21 *	*0.89 *	*8 *	*24.46 ± 0.15 *	*0.60 *	*8 *	*24.35 ± 0.20 *	*0.82 *	*24.35 ± 0.46 *	*1.88 *
	*750 *	*25.18 ± 0.24 *	*0.95 *	*8 *	*24.30 ± 0.12 *	*0.51 *	*8 *	*24.97 ± 0.10 *	*0.42 *	*8 *	*24.82 ± 0.16 *	*0.63 *	*24.82 ± 0.41 *	*1.67 *
	*500 *	*26.66 ± 0.82 *	*3.07 *	*8 *	*25.36 ± 0.23 *	*0.92 *	*8 *	*26.19 ± 0.24 *	*0.92 *	*8 *	*26.07 ± 0.43 *	*1.64 *	*26.07 ± 0.73 *	*2.82 *
	*250 *	*28.66 ± 0.86 *	*3.01 *	*8 *	*28.38 ± 1.91 *	*6.72 *	*8 *	*28.97 ± 0.59 *	*2.05 *	*8 *	*28.67 ± 1.12 *	*3.93 *	*28.67 ± 1.22 *	*4.27 *
	*100 *	*29.17 ± 0.53 *	*1.80 *	*8 *	*27.78 ± 0.49 *	*1.75 *	*8 *	*28.16 ± 0.53 *	*1.89 *	*7 *	*28.37 ± 0.52 *	*1.82 *	*28.38 ± 0.78 *	*2.76 *
	*50 *	*30.95 ± 0.43 *	*1.39 *	*2 *	*29.11 ± 0.67 *	*2.31 *	*4 *	*29.93 ± 1.11 *	*3.70 *	*3 *	*30.00 ± 0.74 *	*2.47 *	*29.79 ± 1.03 *	*3.47 *
	**250**	**26.27 ± 0.26**	**0.99**	**8**	**26.70 ± 0.27**	**1.02**	**8**	**26.67 ± 0.42**	**1.56**	**8**	**26.55 ± 0.37**	**1.19**	**26.55 ± 0.37**	**1.39**
	**100**	**27.22 ± 0.68**	**2.48**	**8**	**28.02 ± 0.39**	**1.38**	**8**	**27.74 ± 0.34**	**1.21**	**8**	**27.66 ± 0.58**	**1.69**	**27.66 ± 0.58**	**2.09**
	**50**	**27.56 ± 0.53**	**1.94**	**8**	**28.26 ± 0.41**	**1.47**	**8**	**28.33 ± 0.40**	**1.41**	**8**	**28.05 ± 0.56**	**1.61**	**28.05 ± 0.56**	**2.00**
	**25**	**27.87 ± 0.69**	**2.47**	**8**	**29.34 ± 0.55**	**1.89**	**8**	**28.58 ± 0.41**	**1.45**	**8**	**28.59 ± 0.82**	**1.94**	**28.59 ± 0.82**	**2.85**
	**10**	**28.75 ± 0.78**	**2.70**	**6**	**29.44 ± 0.55**	**1.86**	**5**	**29.08 ± 0.33**	**1.14**	**7**	**29.07 ± 0.60**	**1.90**	**29.07 ± 0.60**	**2.07**
	**5**	**28.82 ± 0.15**	**0.53**	**4**	**28.71 ± 0.21**	**0.72**	**3**	**28.88 ± 0.32**	**1.12**	**4**	**28.81 ± 0.23**	**0.79**	**28.81 ± 0.23**	**0.79**

The intra- and interassay variability and coefficient of variations were calculated by the formulas: interassay CV = standard deviation of all replicates/(mean Ct-value of all replicates) × 100, intra-assay CV = mean of the CVs of all runs.

^*^Average Ct values: mean ± standard deviation.

^**^CV (%) = standard deviation/mean Ct × 100.

^***^All PCR comparisons were run on the Rotor Gene Q MDx thermocycler (in italic font) and on the Mx3005P thermocycler (in bold).

**Table 6:** Comparison of the 10 commercial master mixes with regard to the practicability of the different assays and the costs, times, and number of pipetting steps.

	Ranking for cost/PCR reaction^*^	Run duration (minutes)		Number of pipetting steps^**^
		Rotor Gene Q MDx	Mx3005P	
Two-step RT-qPCR:				
SsoFast(Iscript)	9	2:42	2:53	12
QuantiTect (QuantiTect)	8	2:54	3:00	14
Maxima (Maxima)	7	2:57	3:08	14
Perfecta (qScript)	6	2:48	2:56	11
Express (SS Vilo)	10	2:50	3:01	10
One-step qRT-PCR:				
Quantitect-1 step	5	2:48	2:57	7
Rotor Gene-1 step	2	2:39	2:48	7
Verso-1 step	3	2:46	2:57	8
SSIII Platinium-1 step	4	2:18	2:28	7
qScript-1 step	1	2:38	2:48	7

^*^The cost was calculated by dividing the cost for each commercial kit, based on the manufacturer's list price in summer of 2013 by the number of PCR reactions that could be performed with each kit. The kits were ranked from 1 to 10 based on their cost per reaction, with 1 corresponding to the least expensive and 10 to the most expensive.

^**^Pipetting steps include those corresponding to the preparation of the master mix and the reverse transcription and PCR steps.

## Abbreviations

Ct: Threshold cycle
CV: Coefficient of variation
EBLV-1: European bat* Lyssavirus* type 1
LOD 95%: Limit of detection at 95%
PCR: Polymerase chain reaction
RT: Reverse transcription
*R*^2^: Coefficient of determination.
